# Somatic mutations and progressive monosomy modify *SAMD9*-related phenotypes in humans

**DOI:** 10.1172/JCI91913

**Published:** 2017-03-27

**Authors:** Federica Buonocore, Peter Kühnen, Jenifer P. Suntharalingham, Ignacio Del Valle, Martin Digweed, Harald Stachelscheid, Noushafarin Khajavi, Mohammed Didi, Angela F. Brady, Oliver Blankenstein, Annie M. Procter, Paul Dimitri, Jerry K.H. Wales, Paolo Ghirri, Dieter Knöbl, Brigitte Strahm, Miriam Erlacher, Marcin W. Wlodarski, Wei Chen, George K. Kokai, Glenn Anderson, Deborah Morrogh, Dale A. Moulding, Shane A. McKee, Charlotte M. Niemeyer, Annette Grüters, John C. Achermann

**Affiliations:** 1 Genetics and Genomic Medicine, University College London (UCL) Great Ormond Street Institute of Child Health, London, United Kingdom.; 2 Institute of Experimental Pediatric Endocrinology and Department of Pediatric Endocrinology, Charité, Berlin, Germany.; 3 Department of Human and Medical Genetics, Charité, Berlin, Germany.; 4 Berlin Institute of Health, Berlin, Germany, and Berlin-Brandenburg Centrum for Regenerative Therapies, Charité, Berlin, Germany.; 5 Department of Paediatric Endocrinology, Alder Hey Children’s NHS Foundation Trust, Liverpool, United Kingdom.; 6 North West Thames Regional Genetics Service, Northwick Park Hospital, Harrow, United Kingdom.; 7 Institute of Medical Genetics, University Hospital of Wales, Cardiff, United Kingdom.; 8 Academic Unit of Child Health, University of Sheffield, Sheffield, United Kingdom.; 9 Department of Endocrinology, Children’s Health Queensland Clinical Unit, University of Queensland, Brisbane, Australia.; 10 Department of Neonatology, University of Pisa, Pisa, Italy.; 11 Pediatric Endocrinology, Karlsruhe, Germany.; 12 Department of Pediatrics and Adolescent Medicine, Division of Pediatric Hematology and Oncology, Medical Center University of Freiburg, Faculty of Medicine, University of Freiburg, Freiburg, Germany.; 13 German Cancer Consortium (DKTK) and German Research Center (DKFZ), Heidelberg, Germany.; 14 Max Delbrück Center for Molecular Medicine, Berlin, Germany.; 15 Department of Paediatric Histopathology, Alder Hey Children’s NHS Foundation Trust, Liverpool, United Kingdom.; 16 Histopathology Department, Great Ormond Street Hospital for Children NHS Foundation Trust, London, United Kingdom.; 17 North East Thames Regional Genetics Laboratory Service, Great Ormond Street Hospital for Children NHS Foundation Trust, London, United Kingdom.; 18 Developmental Biology and Cancer, UCL Great Ormond Street Institute of Child Health, London, United Kingdom.; 19 Department of Genetic Medicine, Belfast City Hospital, Belfast, United Kingdom.

## Abstract

It is well established that somatic genomic changes can influence phenotypes in cancer, but the role of adaptive changes in developmental disorders is less well understood. Here we have used next-generation sequencing approaches to identify de novo heterozygous mutations in sterile α motif domain–containing protein 9 (*SAMD9*, located on chromosome 7q21.2) in 8 children with a multisystem disorder termed MIRAGE syndrome that is characterized by intrauterine growth restriction (IUGR) with gonadal, adrenal, and bone marrow failure, predisposition to infections, and high mortality. These mutations result in gain of function of the growth repressor product SAMD9. Progressive loss of mutated *SAMD9* through the development of monosomy 7 (–7), deletions of 7q (7q–), and secondary somatic loss-of-function (nonsense and frameshift) mutations in *SAMD9* rescued the growth-restricting effects of mutant SAMD9 proteins in bone marrow and was associated with increased length of survival. However, 2 patients with –7 and 7q– developed myelodysplastic syndrome, most likely due to haploinsufficiency of related 7q21.2 genes. Taken together, these findings provide strong evidence that progressive somatic changes can occur in specific tissues and can subsequently modify disease phenotype and influence survival. Such tissue-specific adaptability may be a more common mechanism modifying the expression of human genetic conditions than is currently recognized.

## Introduction

Sterile α motif domain–containing protein 9 (SAMD9, OMIM 610456) is a 1,589–amino acid protein that is encoded by a gene on the long arm of chromosome 7 (7q21.2) (1). SAMD9 has little homology to other proteins except for SAMD9L, also on 7q21.2, with which it shares 58% identity (1–3). SAMD9 and SAMD9L are likely to act as growth suppressors, and overexpression of SAMD9 suppresses tumor progression in non–small cell lung cancer cells (4). In contrast, reduced SAMD9 expression has been reported in several tumor tissues, cisplatin chemoresistance, and normophosphatemic familial tumoral calcinosis (5–7). Similarly, loss of *SAMD9* and *SAMD9L* by monosomy 7 or monosomy 7q (–7/7q–) is well established in myeloid lineage malignancies (8, 9), and disruption of SAMD9L has been shown to be associated with the development of myelodysplastic syndrome (MDS) in mice and humans (10, 11). These growth-regulating effects are potentially mediated by altered endosomal degradation of the EGF receptor (EGFR) (10).

Three case reports of children with intrauterine growth restriction (IUGR), congenital adrenal insufficiency, gonadal failure, and monosomy 7 have been published in the last 20 years (12–14). These children also developed MDS, presumably secondary to the monosomy 7, but the interrelation of these features was not understood.

Very recently, heterozygous gain-of-function mutations in *SAMD9* have been reported in 11 patients with IUGR, adrenal insufficiency, and gonadal failure, together with bone marrow failure (15). Two of these patients had monosomy 7 and developed MDS. This condition has been termed MIRAGE syndrome (myelodysplasia, infections, restriction of growth, adrenal hypoplasia, genital phenotypes, enteropathy) (OMIM 617053). Through different next-generation sequencing approaches we have simultaneously identified *SAMD9* mutations in 8 patients with a complex multisystem growth restriction phenotype. Here we are able to show, for the first time to our knowledge, that complex dynamic somatic changes in *SAMD9* and the *SAMD9*/*SAMD9L* locus on chromosome 7q occur, which are associated with this distinct disease phenotype and modify survival in these patients.

## Results

### SAMD9 mutations cause a multisystem growth restriction disorder.

**Figure 1:**
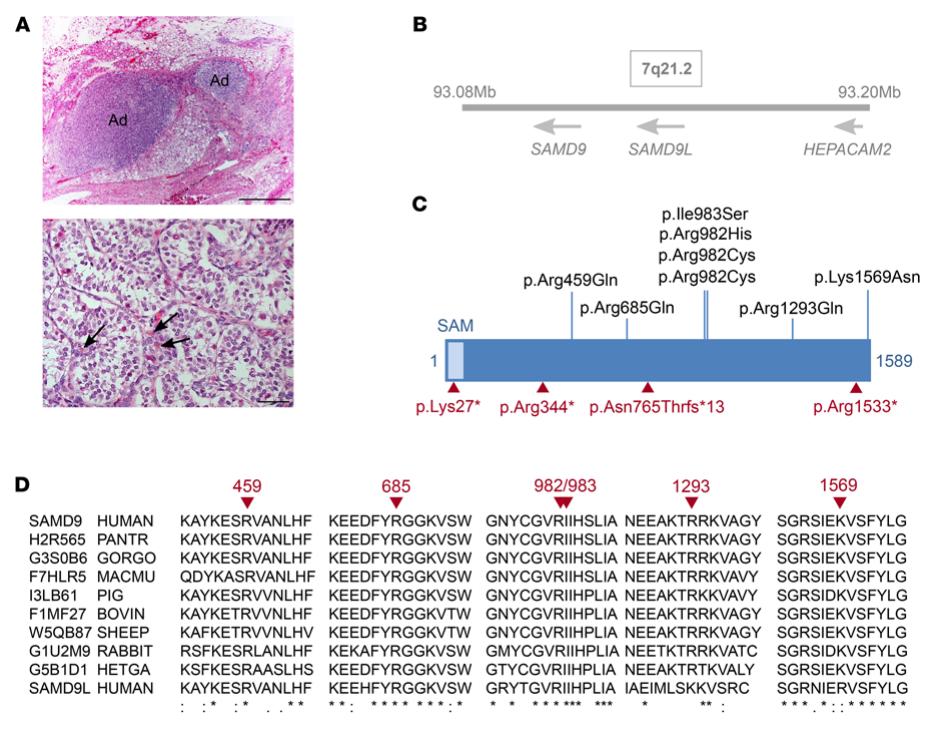
Mutations in SAMD9 identified in 8 patients with IUGR and a multisystem disorder. (**A**) Adrenal and testis histology from patient 4. Top: Small islands of adrenal tissue (Ad) were identified in perinephric fat. Scale bar: 1 mm. Bottom: Testis showing rare Leydig cells (arrows) between seminiferous cords. Scale bar: 100 μm. (**B**) The location of *SAMD9*, *SAMD9L*, and *HEPACAM2* on chromosome 7q21.2. (**C**) Mutations resulting in increased growth repression are shown in black. Acquired somatic loss-of-function mutations are shown in red. (**D**) Conservation of SAMD9 protein sequence in different species, with the position of mutated residues shown in red. Human SAMD9 is shown on top. Human SAMD9L is shown at the bottom.

Eight patients with marked IUGR and severe testicular dysfunction leading to female external genitalia in six children and atypical (“ambiguous”) external genitalia in 2 children were referred to our centers for treatment and precise genetic analysis (Table 1). Testes were undescended in most (7 of 8) patients with preserved structure but decreased testosterone, where assessed (Figure 1A and Supplemental Tables 1 and 2; supplemental material available online with this article; https://doi.org/10.1172/JCI91913DS1). Neonatal-onset severe adrenal insufficiency was diagnosed in 6 of 8 children, whereas 1 had mild adrenal dysfunction and another had normal adrenal function into adolescence (Figure 1A and Supplemental Tables 1 and 2). All children were delivered preterm and were severely ill in the neonatal period and needed intensive care. Other features included severe pulmonary infections and a bronchopulmonary dysplasia–like phenotype requiring oxygen, recurrent and severe bacterial and viral infections, persistent diarrhea, severe diaper rashes, frontal bossing, hydrocephalus due to membrane development induced by infections, and bone marrow failure (thrombocytopenia, anemia) (Table 1). Six of the children died in the first 2 years of life. Two children (patients 7 and 8) survived but developed MDS with monosomy 7 and received bone marrow transplantation (Table 1). Following written informed consent from families, DNA samples were obtained from all patients, fibroblasts from 3 of them, and bone marrow cells from 2 to try to identify the underlying basis of this condition.

Using a combination of whole-exome sequencing and targeted capture, heterozygous *SAMD9* mutations were identified in all 8 children (Table 1 and Figure 1, B and C). These changes were de novo in all 7 children in whom parental DNA was available and were located in highly conserved residues, often affected arginines (6 of 8) with half of all mutations clustering in a hotspot of codons 982/983 (Figure 1, C and D). Although *SAMD9* has many single nucleotide variations, the specific mutations identified in these children were not found in more than 100,000 alleles in public databases nor in more than 100 children with primary adrenal insufficiency and normal birth weight. Moreover, the mutations in the patients show clustering in a key hotspot, which is often reported in gain-of-function changes (16).

**Figure 2:**
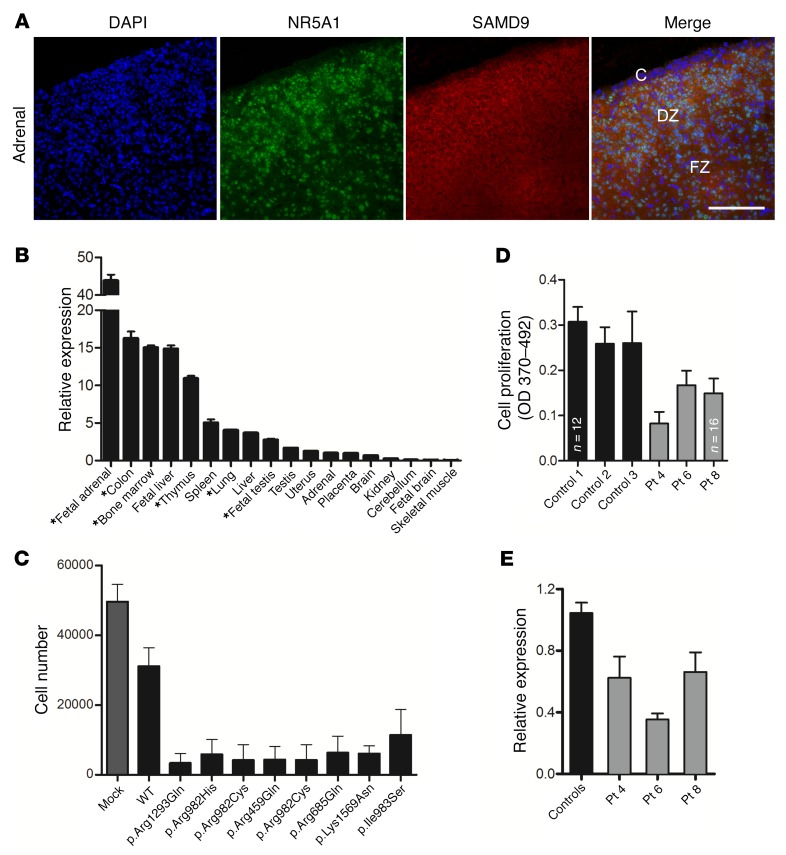
SAMD9 is expressed in key tissues and represses cell growth. (**A**) Immunohistochemistry showing SAMD9 expression in human fetal adrenal gland at 9 weeks postconception (red) with NR5A1 (also known as steroidogenic factor 1) shown in green and DAPI-stained nuclei in blue. C, capsule; DZ, definitive zone; FZ, fetal zone. Scale bar: 100 μm. (**B**) *SAMD9* expression in different human fetal and adult tissues showing high expression in fetal adrenal as well as in tissues affected in the clinical phenotype. Tissues especially relevant to the phenotype are highlighted (*). Data are shown as relative expression compared with *GAPDH*. Representative data are shown as mean ± SEM for a single study performed with triplicate technical replicates. Similar patterns were seen in independent studies using both *GAPDH* and *ACTB* as housekeeping genes. (**C**) Growth of HEK293 cells was reduced following stable transfection of WT SAMD9 (mean ± SD; mock vs. WT, *P* < 0.001) and reduced further by the gain-of-function mutations found in patients (WT vs. samples, *P* < 0.0001, black bars). Data represent mean ± SD for the absolute cell number in 3 independent studies, each performed in triplicate (*n* = 9; 2-tailed *t* tests). (**D**) BrdU assays showing significantly reduced growth of patient fibroblasts (patients 4, 6, and 8) compared with fibroblasts from 3 independent controls. Data represent mean ± SD cell proliferation for 3 independent studies, each with 6 technical replicates (unless specified, *n* = 18; 1-way ANOVA with Tukey’s multiple comparison test, controls vs. patients, *P* < 0.001). (**E**) qRT-PCR analysis of fibroblast samples from 3 patients with a *SAMD9* gene mutation revealed a significant reduction of gene expression compared with *SAMD9* expression in fibroblasts derived from 3 healthy control individuals. Data represent mean ± SEM relative expression for 4 independent studies, each with 2 replicates (Mann-Whitney test, controls vs. patients, *P* < 0.05).

Immunohistochemistry of human fetal adrenal tissue showed generalized localization of SAMD9 in the cytoplasm of cells in the definitive and fetal zones, with strong expression in cells positive for the nuclear receptor NR5A1 (also known as steroidogenic factor 1, or SF-1) (Figure 2A). In the testis, SAMD9 was present in interstitial (Leydig) cells and seminiferous cords (Supplemental Figure 1). Using quantitative reverse transcriptase PCR of human fetal and adult tissues, *SAMD9* expression was highest in the fetal adrenal gland, with high expression also in the colon, bone marrow, fetal liver, thymus, spleen, lung, liver, and fetal testis (Figure 2B). This expression pattern correlates very well with the clinical phenotype seen in the patients (Table 1).

### SAMD9 is a growth repressor, and SAMD9 mutations further reduce cell proliferation.

Stable selection of HEK293 cells transfected with SAMD9 mutant constructs was undertaken and cell proliferation studied. Transfection of WT SAMD9 resulted in decreased cell proliferation, consistent with a role for SAMD9 as a growth repressor (Figure 2C). Transfection of SAMD9 harboring the mutations found in our patients resulted in further repression of cell proliferation, consistent with the variants having a gain-of-function effect (Figure 2C). Cell proliferation assays were also performed in fibroblasts from 3 patients. These fibroblasts express the mutated alleles and showed significantly decreased proliferation in comparison with control cells (Figure 2D). Additionally, we analyzed the SAMD9 protein and mRNA expression in fibroblast samples of these 3 patients and identified a reduced expression pattern compared with that of samples from 3 healthy control individuals (Figure 2E and Supplemental Figure 2).

### Progressive loss of chromosome 7 occurs in patients surviving beyond early infancy.

**Figure 3:**
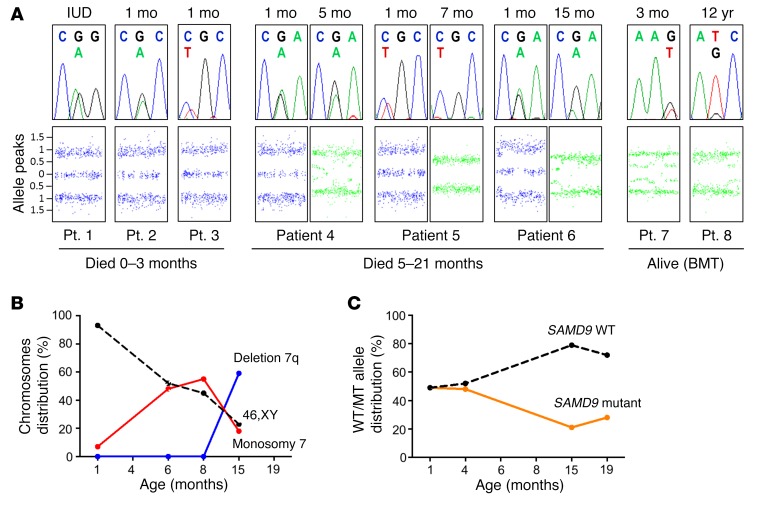
Acquired loss of chromosome 7 (–7) and its long arm (7q–) occurred in association with a reduction in the gain-of-function SAMD9 mutations. (**A**) Chromatograms (top) from all 8 patients showing the *SAMD9* mutations and a reduction in the mutant peak following development of monosomy 7 as shown by cytogenomic array (bottom). All samples are from peripheral leukocytes except the later sample in patient 4, which is from bone marrow DNA. Typical disomic chromosome 7 signal is shown in blue, whereas the signal with monosomy is shown in green. Data are presented in relation to length of survival. For patients 4–6 the earlier array is shown on the left and the later array on the right. The age at sampling is shown above the figure. BMT, bone marrow transplantation; IUD, intrauterine death; mo, months. (**B**) Serial cytogenetic data (leukocytes) from patient 6 showing the expansion of cells with –7 followed by their displacement by a 7q– clone removing the locus containing the *SAMD9* gene. (**C**) Serial changes in the WT and mutant allele percentage (%) in patient 6 show that –7/7q– is associated with a reduction of the mutant *SAMD9* allele, most likely due to the growth selection advantage of bone marrow cells that have lost the growth-repressing mutation. Data are derived from single-nucleotide primer extension assays for leukocyte DNA. A similar reduction in mutant allele was found in other patients who developed –7/7q– (patients 4 and 5) or who presented with a –7/7q– clone at the time of diagnosis of MDS (patients 7 and 8) (Table 1 and Supplemental Figures 3 and 4).

Follow-up of patients surviving the first 3 months of life showed that all of them had evidence of monosomy 7 (–7) or partial deletions of its long arm (7q–) (Table 1 and Figure 3A). Most importantly, by analyzing serial samples available in 3 patients by whole-genome array, we could demonstrate that –7/7q– was not present at early time points but these changes were acquired later (Figure 3A; patients 4–6). Furthermore, in the 2 patients with MDS at the time of diagnosis, significant –7/7q– ratios were detected (patients 7 and 8). By studying chromatogram profiles, single-nucleotide primer extension assays, and DNA subcloning, we showed that the development of –7 removed the allele harboring the gain-of-function *SAMD9* mutation (Table 1; Figure 3, A–C; and Supplemental Figures 3 and 4). Furthermore, in patient 6, bone marrow cells with –7 were predominant initially but were later replaced by cells with 7q–, resulting in more specific removal of the locus containing the mutated *SAMD9* (Figure 3B).

### Demonstration of a dynamic evolution of secondary loss-of-function mutations in SAMD9 that rescue growth repression.

**Figure 4:**
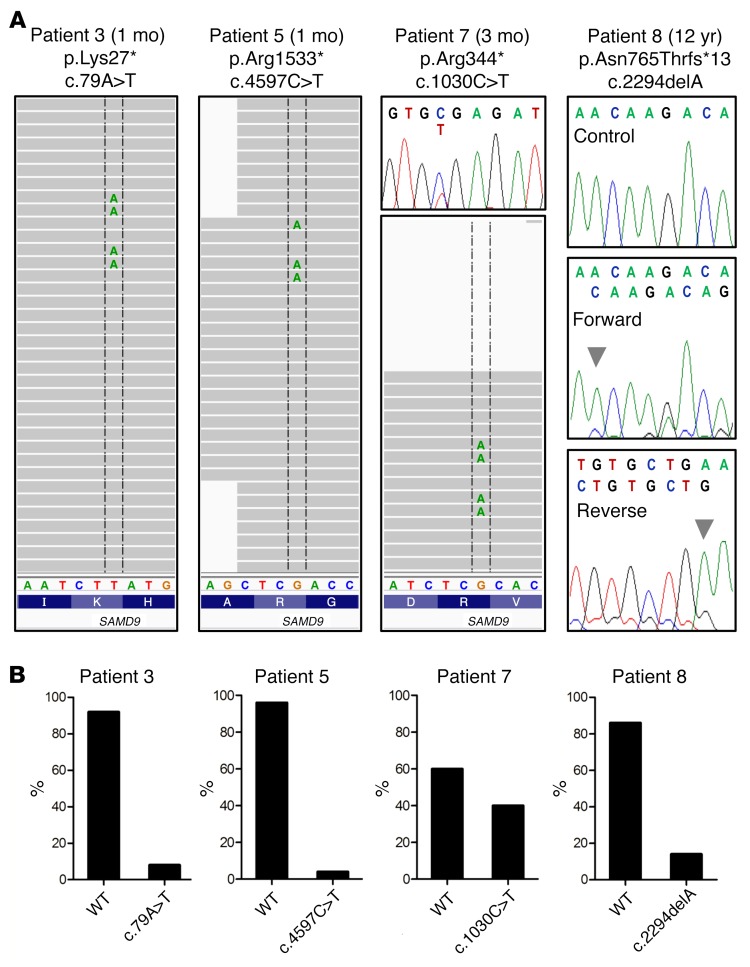
Additional loss-of-function mutations occurring in SAMD9. (**A**) Low-copy-number nonsense (stop gain) mutations were found in 2 infants who died in the first year of life (patients 3 and 5). These changes were not seen on chromatograms but were detected by deep sequencing and by subcloning of DNA amplicons and sequencing of 50–100 clones. Both patients with a milder phenotype (patients 7 and 8) had loss-of-function mutations detected on chromatograms. A single nucleotide variant causing a nonsense mutation was found in patient 7 (chromatogram and deep sequencing reads shown). A single nucleotide deletion (arrowhead) causing a frameshift was found in patient 8. Control and patient forward sequence is shown in the upper panels and the patient’s reverse sequence shown below. Note: The reverse-direction nucleotide sequence is shown in the next-generation sequencing reads. Patient age at sampling is indicated. (**B**) Single-nucleotide primer extension assays confirming the WT and mutant allele percentage of additional *SAMD9* loss-of-function changes in the patients described above.

**Figure 5:**
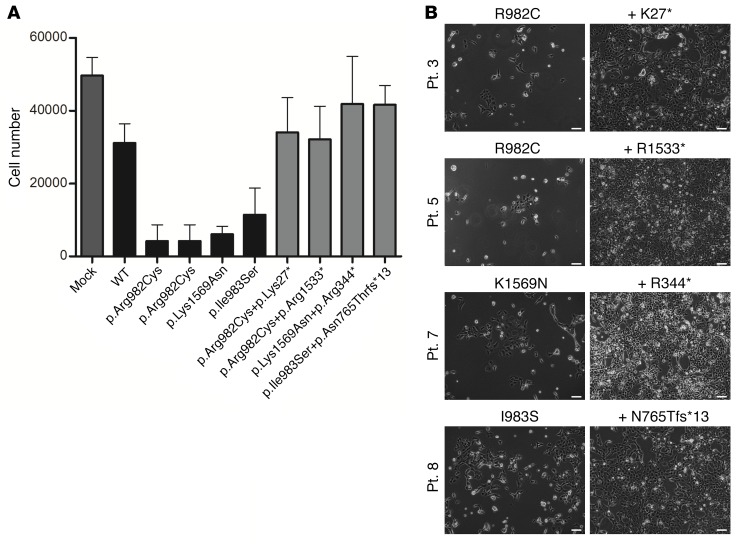
Secondary loss-of-function mutations “rescue” the growth-repressive effects of SAMD9 gain-of-function mutations. (**A**) The effects of the naturally occurring secondary nonsense or frameshift changes in the SAMD9 protein were studied in HEK293 cells in the same experiments as shown in Figure 2C. Addition of these secondary changes resulted in a loss of repressor activity (primary mutant vs. double mutant, all *P* < 0.0001, dark gray bars). Data represent mean ± SD for the absolute cell number in 3 independent studies, each performed in triplicate (*n* = 9; 2-tailed paired *t* test). (**B**) Representative images of cell density for the primary mutation (left column) and combined primary and secondary mutations (right column) for patients 3, 5, 7, and 8. Scale bars: 100 μm.

In addition to the chromosomal changes identified, 4 children had evidence of somatic nonsense (stop gain) or frameshift (single nucleotide deletion) loss-of-function mutations in *SAMD9* in their hematopoietic compartment (Table 1 and Figure 1C). Two of these changes were not detected using standard Sanger sequencing, but were present in a proportion of deep sequencing reads (Figure 4A). All changes were confirmed and quantified using single-nucleotide primer extension assays and by subcloning and sequencing of PCR-amplified DNA (Figure 4B and Supplemental Figures 5 and 6). Functional studies were performed in HEK293 cells transfected with *SAMD9* containing the primary gain-of-function mutant together with the relevant loss-of-function change. These cells showed increased proliferation, suggesting that the growth-repressing effects of gain-of-function mutations in SAMD9 can be “rescued” by these secondary loss-of-function changes (Figure 5, A and B).

These data show that the expression of the mutant SAMD9 protein was abrogated in most children by more than 1 mechanism. In patients 5, 7, and 8, –7/7q– occurred together with a loss-of-function point mutation (Table 1). In patient 6, bone marrow cells showed progressive monosomic loss of the *SAMD9* locus (Figure 3, B and C).

### SAMD9 mutations produce subtle changes in early endosome size.

**Figure 6:**
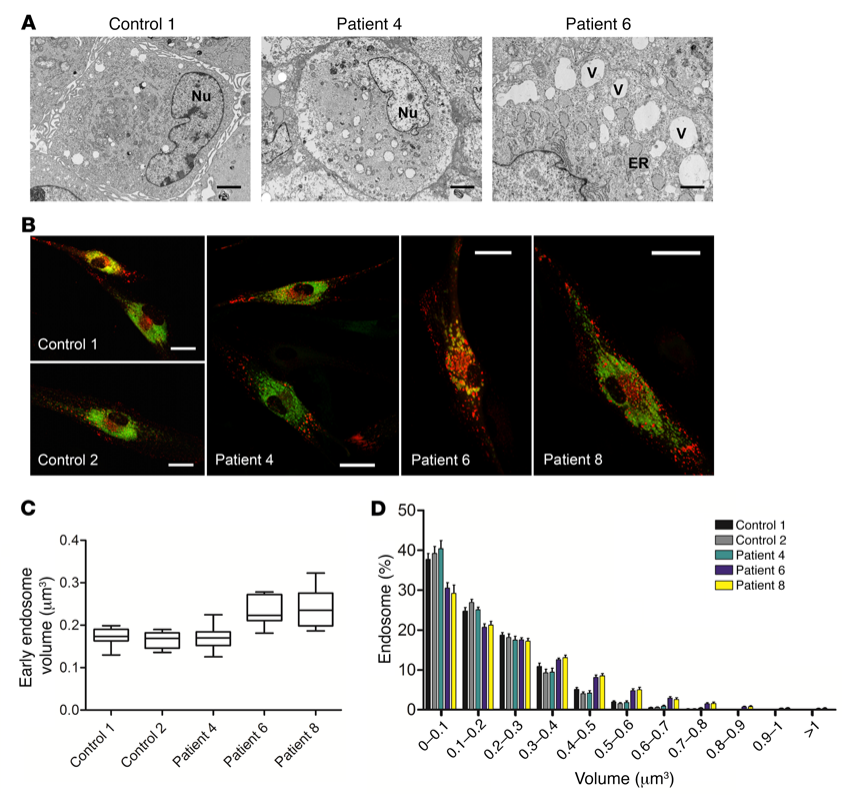
Analysis of the effect of SAMD9 mutations on cell structure and endosomes. (**A**) Electron microscopy of fibroblasts from controls and patients showed a modest increase in the number and size of cytoplasmic vesicles (V) and mildly dilated rough endoplasmic reticulum (ER) in a small subpopulation of patient cells, but the effects were very heterogeneous (see also Supplemental Figure 7). Nu, nucleus. Scale bars: 2 μm. (**B**) Patient fibroblasts showing early endosomes (Rab5a, red) and late endosomes (Rab7a, green). Scale bars: 10 μm. (**C**) Early endosome volumes were greater in fibroblasts from patients 6 and 8 compared with controls, with greater variability in patients’ samples. Mean endosome volume for 10 independent cells was analyzed for each group. Data are shown as mean values with a whiskers plot to show the range (1-way ANOVA and Tukey’s post hoc test, *P* < 0.05). (**D**) A distribution analysis of early endosome volume for all cells studied also showed a shift toward larger early endosome volume for patients 6 and 8. Data are shown as mean ± SEM for 10 independent cells in each group. Mean number of endosomes quantified per cell, *n* = 2,248.

Electron microscopy of patient fibroblasts compared with control cells showed that a small subpopulation of patient cells contained multiple large vesicles (>1.5 μm) as well as dilated endoplasmic reticulum and enlarged mitochondria, but these changes were heterogeneous, and extensive areas of cytoplasm containing large vesicles were not seen (Figure 6, A and B, and Supplemental Figure 7). No marked differences in late endosome structure were seen between patient and control fibroblasts (Figure 6B, green structures). A modest increase in the size of early endosomes (red structures) was identified in 2 of the patient cell lines (patients 6 and 8), as shown in representative images (Figure 6B) and in quantitative analysis of early endosome size (Figure 6, C and D).

### Induced pluripotent stem cells containing SAMD9 mutations can be differentiated into intermediate mesoderm.

**Figure 7:**
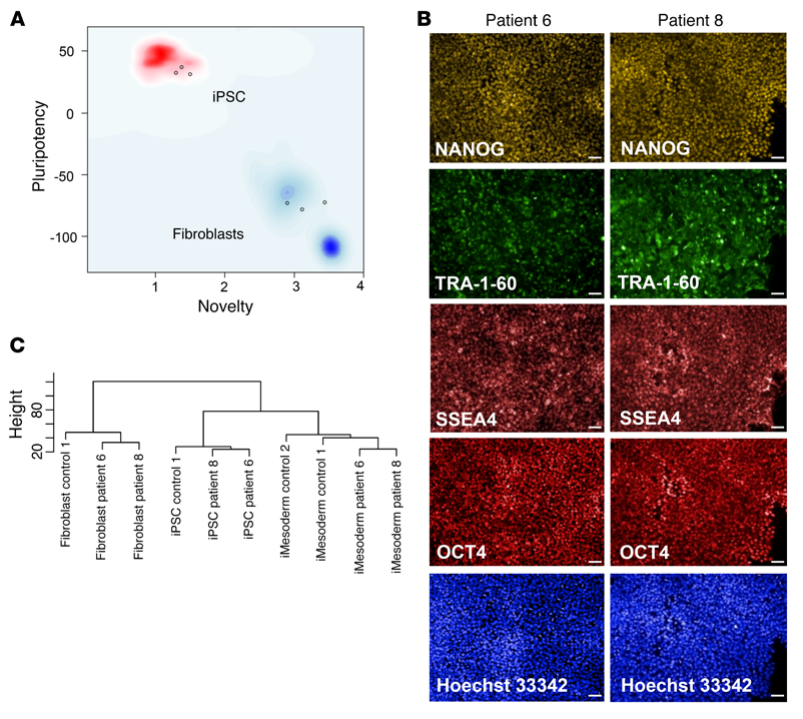
Effects of SAMD9 mutations on the derivation of fibroblasts into iPSCs and further differentiation into intermediate mesoderm. (**A**) PluriTest analysis of the array data. Undifferentiated patient-derived iPSCs fall within the empirically set thresholds for pluripotent cells. The plot shows that the 2 patient lines (patients 6 and 8) and the normal control iPSC line cluster with pluripotent stem cells (red cloud) in contrast to the fibroblasts they originated from that cluster with partly reprogrammed or differentiated cells (blue clouds). Each circle represents 1 iPSC or fibroblast line. (**B**) iPSCs of both patients stained positive for the pluripotency markers NANOG, tumor rejection antigen 1-60 (TRA-1-60) (cell surface), stage-specific embryonic antigen 4 (SSEA4) (cell surface), and OCT3/4 (nuclear). Nuclei were counterstained with Hoechst 33342. Scale bars: 50 μm. (**C**) Hierarchical clustering of patient and control samples showing clustering by cell type (fibroblasts, iPSCs, intermediate mesoderm). Within each cell group, patient samples containing SAMD9 mutations grouped together. Functional enrichment analysis using DAVID showed potential changes in biologically relevant pathways in the 2 patient samples compared with control samples in the different cell lines (Supplemental Table 3).

In order to determine the effects of SAMD9 mutants on cellular differentiation and function, induced pluripotent stem cells (iPSCs) were generated from fibroblasts of 2 of the patients (patients 6 and 8). Cells harboring the primary gain-of-function *SAMD9* mutations showed iPSC differentiation characteristics similar to those of controls based on pluripotency tests, stem cell markers, and hierarchical clustering of RNA expression data (Figure 7, A–C, and Supplemental Figure 8). Cells could be differentiated into intermediate mesoderm with profiles similar to those of control cells (Figure 7C). Gene expression analysis of the cells followed by hierarchical clustering showed that patient-derived cells (fibroblasts, iPSCs, intermediate mesoderm) did cluster with each other and were distinct from control cells (Figure 7C). This finding likely represents an effect of the underlying SAMD9 mutation, potentially influencing EGFR signaling and other biologically relevant pathways (e.g., urogenital development) (Supplemental Table 3). Interestingly, apoptotic and inflammatory signals seem to be deregulated during mesoderm differentiation, suggesting that, in addition to defective proliferation, excessive stress signals might also contribute to hematopoietic failure.

## Discussion

Using a combination of whole-exome sequencing and targeted capture, we have identified a severe multisystem disorder caused by heterozygous *SAMD9* mutations in eight 46,XY patients with marked IUGR, severe testicular dysfunction, congenital adrenal insufficiency, thrombocytopenia/anemia, persistent diarrhea, and severe infections resulting in a life-threatening course. Three children died in utero or in the first 3 months of life, 3 children died between 3 months of age and 2 years, and 2 children were diagnosed with MDS and survived following bone marrow transplantation. Detailed studies in a panel of fetal and adult tissues show that *SAMD9* expression closely mirrors the features of the clinical syndrome.

An independent study has very recently reported a similar, though distinct, phenotype caused by *SAMD9* mutations (15), characterized by IUGR, infections, enteropathy, adrenal hypoplasia, and underdeveloped external genitalia (MIRAGE syndrome) (15). Only 2 of the 11 patients reported had monosomy 7 and developed myelodysplasia. In contrast, all children in our study had a 46,XY karyotype, and significantly more marked gonadal dysfunction with female-appearing external genitalia in 6 of them, as well as other additional features such as hydrocephalus, cerebellar hypoplasia, small thymus, and short phalanges, and severe neonatal disease with higher mortality in early infancy. Therefore, this cohort represents a significantly different subset of the phenotypical spectrum. Although a small increase in early endosome size was seen in fibroblasts from 2 of our patients, as well as some enlarged vesicles and organelles, we were unable to detect the large late endosomes or large destructive vesicles described in the other report (15).

Whereas only 2 of the 11 patients reported by Narumi et al. had evidence of mosaic monosomy 7, 6 of the 8 patients in our cohort had evidence of complex and dynamic somatic events in hematopoietic cells that would be predicted to abrogate the growth-repressing gain-of-function mutations in *SAMD9*.

Using several different approaches, it was possible to show consistently that the development of –7 removed the allele harboring the *SAMD9* mutation. This has been termed “adaption by aneuploidy,” but examples of this phenomenon influencing disease mechanism in humans are so far limited to SAMD9/SAMD9L (11, 15). For the first time, to our knowledge, we are now able to show that the development of –7/7q– was progressive in 3 patients, with 1 child (patient 6) showing even complex dynamic cytogenetic changes involving first –7 and then 7q– over time.

Furthermore, 4 children additionally had evidence of the development of somatic nonsense (stop gain) or frameshift (single nucleotide deletion) mutations in *SAMD9* in their hematopoietic compartment. In the 2 children with the mildest phenotype, these somatic changes could be detected by traditional Sanger sequencing. However, 2 other children had evidence of second somatic loss-of-function changes that were only detected by deep sequencing and confirmed by single-nucleotide primer extension assays or by subcloning of PCR-amplified DNA. These changes would not have been detected by traditional sequencing approaches.

As a consequence of these events, the expression of the mutant SAMD9 protein progressively became abrogated in most children by more than 1 mechanism. These observations indicate that bone marrow cells that lost the mutated *SAMD9* allele had a clonal advantage. The coexistence of different second-hit mutations all affecting the mutated *SAMD9* allele strongly suggests that the putative gain-of-function germline mutation imposed severe growth restriction on the hematopoietic system reflected by the clinical symptoms of the patients, who suffered from anemia, thrombocytopenia, and infections from early infancy. As a consequence, the pressure of cells to lose the mutated *SAMD9* gene seemed to be exceptionally high. The genetic heterogeneity of bone marrow cells found in patients 5, 6, 7, and 8 gave evidence that a second hit occurred early and simultaneously in different hematopoietic stem and progenitor cells, all giving rise to clones with increased competitiveness (–7/7q–). However, this phenomenon was associated with the risk of malignant transformation. It has to be assumed that MDS in these patients was polyclonal rather than monoclonal, at least during the initial disease stages. Under certain circumstances and depending on the additional somatic hits, 1 single clone might have overgrown the others, as observed for the 7q– clone in patient 6. Therefore, the detection of multiple genomes in an individual might not only represent randomly occurring events, but could also reflect clonal expansion of cells with deletions and loss-of-function mutations that have a selection and proliferation advantage over cells carrying deleterious gain-of-function changes.

The outcome (length of survival) seemed to correlate with the progressive development of –7/7q– and of second hits in the bone marrow. Notably, the 2 children (patients 7 and 8) with the mildest phenotypes had non-arginine primary mutations, large clones with –7 or 7q– present at time of diagnosis of MDS, and the highest somatic load of the additional nonsense or frameshift change. Therefore, –7 and the other second-hit changes affecting the *SAMD9* locus provided a partial rescue of growth suppression, but paved the way for malignant transformation again limiting survival. Early detection of *SAMD9* mutations in patients with IUGR, adrenal and gonadal failure, and severe neonatal infections is important, because bone marrow transplantation may be necessary for survival. As all of the children in our study who could be tested (7 of 8) had de novo changes, it seems likely that recurrence would only occur if a parent carried a germline mosaicism. This has been reported to be the case in 1 patient (15). Furthermore, *SAMD9* has many single-nucleotide polymorphisms. Some of these have been shown to have no functional consequence by Narumi et al. (15), so careful analysis of potential gain of function will be required.

Although the association between childhood MDS and –7/7q– is well established, the molecular basis of this condition has until recently not been well understood, as many candidate tumor suppressor genes have been identified on chromosome 7, and functional losses of these genes have been implicated in disease initiation and/or progression (9, 17). One putative tumor suppressor is the homolog of *SAMD9*, *SAMD9L*. *SAMD9L* and *SAMD9*, together with the *HEPACAM2* gene, are located on 7q21.1 (Figure 1B), a region that has been found to be deleted in a subset of patients with myeloid leukemia and myelodysplastic syndromes (9). Although targeted deletion of *Samd9l* in the mouse results in myeloid neoplasia, the exact role of SAMD9L in transformation is still unclear (10). Very recently, heterozygous mutations causing a putative gain of function of the growth-suppressing effects of *SAMD9L* have been reported in human ataxia-pancytopenia syndrome (11). Two of these children developed myeloid malignancies, with evidence of monosomy 7 in the bone marrow or in derived cell lines from several of these patients. Therefore, a very similar mechanism to that for SAMD9 is emerging, although it is so far not possible to define whether the primary cause of the malignant transformation is *SAMD9L*, *SAMD9*, or other genes in the locus.

In addition to the dynamic somatic changes, genomic regulation of *SAMD9* may also be important in controlling the balance of cell proliferation. By studying patient fibroblasts compared with controls, we were initially surprised to detect reduced SAMD9 expression at both the RNA and protein levels. This finding may represent reduced transcriptional regulation of *SAMD9* in the context of a gain-of-function change. This hypothesis is supported by the report of *increased* *SAMD9* mRNA in fibroblasts from patients with normophosphatemic familial tumoral calcinosis, a condition that occurs due to loss of SAMD9 function (7). Indeed, previous work has suggested that the *SAMD9* promoter can be regulated by inflammatory cytokines such as TNF-α and IFN-γ (18, 19), so genomic regulation with possible feedback mechanisms might also serve to regulate cellular SAMD9 activity in the context of gain- or loss-of-function changes.

In order to study the underlying pathogenic basis of this condition further, the effects of SAMD9 gain of function on the derivation of iPSCs from fibroblasts, and subsequent differentiation of iPSCs into intermediate mesoderm, were studied in 2 of the patients (patients 6 and 8). iPSCs and intermediate mesoderm were successfully generated, suggesting that SAMD9 does not have a marked effect on early differentiation events. However, the patient samples did cluster together compared with the controls, suggesting changes in gene expression that may involve biologically relevant pathways. Indeed, RNA expression data revealed potential changes in developmental programs, general transcription, and apoptotic and inflammatory signals, all pathways that possibly contribute to the developmental and bone marrow failure phenotype. Even though our results are preliminary, exploiting iPSC technology will provide potentially important disorder- and patient-specific model systems for further studying the mechanisms of developmental conditions. Future studies to try to differentiate intermediate mesoderm, for example into adrenal-like cells, might be informative in revealing SAMD9-related effects, and potentially to study the development and effects of dynamic secondary somatic changes in a relevant in vitro model system.

Based on our observations, SAMD9 is a growth-restricting protein that plays a major role in the development of many systems and leads to intrauterine growth retardation but has a marked effect on adrenal gland and testes differentiation. A similar growth restriction condition causing adrenal hypoplasia is IMAGe syndrome, due to gain-of-function changes in the paternally imprinted cell cycle regulator CDKN1C (20). Children with IMAGe syndrome also have prenatal growth restriction and adrenal insufficiency, but the gonadal phenotype seems less severe, and we are unaware of features such as enteropathies, bone marrow failure, or infections.

Immunohistochemistry of human fetal adrenal gland and testis showed widespread localization of SAMD9 in the cytoplasm of interstitial (Leydig) cells and seminiferous cords, as reported in adults (http://www.proteinatlas.org/ENSG00000205413-SAMD9/tissue) (21). In the testis, SAMD9 may have a greater effect on fetal Leydig cells during the critical early stages of development (6–12 weeks postconception) than Sertoli cells, as testis structure and morphology was relatively well preserved, most children had regression of Müllerian structures (which is dependent on anti-Müllerian hormone from Sertoli cells), and testosterone concentrations were generally lower than expected. The predominant fetal Leydig cell features are similar to the phenotype most commonly associated with disruption of NR5A1, which colocalizes with SAMD9 in many cells (22).

**Figure 8:**
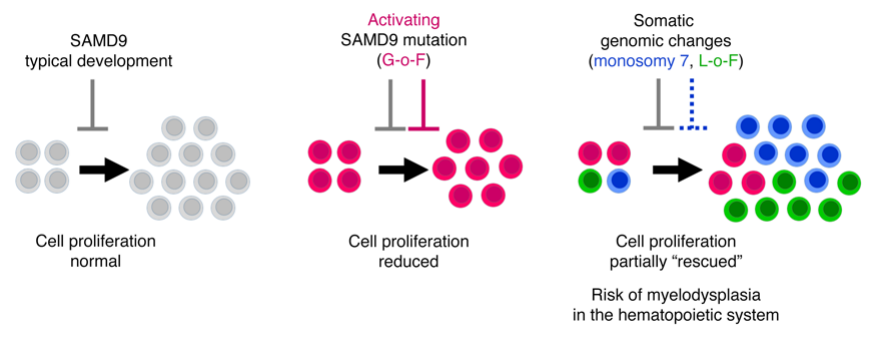
Schematic diagram showing the effects of SAMD9 on cell proliferation during development. During typical development, SAMD9 is a repressor that regulates controlled proliferation of cells (gray). Activation/gain-of-function (G-o-F) mutations in SAMD9 (pink) result in reduced proliferation of cells prior to differentiation, causing tissue hypoplasia. Secondary somatic changes in SAMD9 such as monosomy 7 (blue) or loss-of-function (L-o-F) mutations (green) remove the deleterious effect and allow partial rescue.

It is also emerging that during embryogenesis a fine balance between cell number and differentiation of cells is a prerequisite for normal organ development and size. Several models have been proposed for the adrenal gland and testis, whereby adequate proliferation of cells must occur prior to differentiation (23–26). Organ hypoplasia could result if inadequate proliferation occurs prior to differentiation, or if differentiation occurs too rapidly without sufficient proliferation or outside a critical window. We propose that SAMD9 is essential for controlling cell proliferation to maintain this balance (Figure 8). Gain-of-function mutations in SAMD9 might lead to a significant reduction of cells that could subsequently undergo differentiation/hyperplasia. In this context it is important that SAMD9 has been lost during evolution in the mouse (5). We speculate that this may be one reason why mice are more resistant than humans to the disruptive effects of developmental regulators such as NR5A1 and DAX-1 (also known as NR0B1). Haploinsufficiency of NR5A1 and hemizygosity of DAX-1 cause profound gonadal and adrenal phenotypes, respectively, in humans, but mice do not show such marked features (22, 27–29). Thus mice may be able to adapt in these situations, as they do not have the growth-restrictive effects of SAMD9.

In addition to shedding light on SAMD9 and growth, this study clearly demonstrates, for the first time to our knowledge, that dynamic changes within the genome can influence developmental mechanisms, phenotypes, and even outcome. More than half of the patients studied had evidence of chromosomal changes of the relevant genomic region and/or second mutations in the gene, with complex changes far beyond just “adaption by aneuploidy.” While somatic variability and adaptation are well studied in cancer, there is considerably less evidence to date for these changes influencing development and developmental disorders. Such somatic rescue mechanisms may be most evident in trophic systems with rapid turnover, such as the hematopoietic system. Here, we were able to detect these dynamic changes by analyzing serial DNA samples from leukocytes. However, other dynamic systems might be subject to similar rescue mechanisms, but these changes would never be detected unless serial tissue samples were studied, which, in humans, is often challenging. For example, the absence of an adrenal phenotype in patient 8 may have been due to an adrenal-specific rescue, but obtaining tissue for deep sequencing is not possible. Furthermore, in the presence of aneuploidy, evidence for the primary genetic “driver” may be lost, especially if Sanger sequencing is used. Consequently, dynamic somatic variation of the genome could be present more frequently than appreciated and might contribute to the variable penetrance of phenotypes and even reversibility of certain disorders at a tissue level. Random loss-of-function events might be especially common modifiers when the primary driver is a gain-of-function change, such as shown here for SAMD9.

## Methods

### Patients and samples.

DNA samples were obtained with permission from children referred primarily for the diagnosis of congenital adrenal insufficiency or morphological adrenal abnormalities. One baby had died in utero, and 1 child presented late and was included given the similarity of the phenotype and presence of monosomy 7. DNA samples were obtained from blood leukocytes at different time points using standard methods. Where documented in the article, DNA was also extracted from liver (postmortem), bone marrow, or fibroblasts. Parental DNA samples were obtained from blood or saliva for 7 of 8 children. The clinical characteristics of the cohort are shown in Table 1. Biochemical, endocrine, and cytogenetic investigations were undertaken using standard clinical protocols, and histology of bone marrow, adrenal glands, gonads, and other tissues was confirmed by pathological review.

### Whole-exome sequencing and analysis.

Five micrograms of genomic DNA (gDNA) was enriched using the Agilent Human All Exon V4 kit (Agilent Technologies) following the manufacturer’s protocol. Whole-exome libraries were sequenced on an Illumina HiSeq 2000 system for 1x101 cycles following the manufacturer’s instructions (Illumina). All raw sequencing reads were mapped onto UCSC hg19 using BWA 0.5.9-r16, and mappings were converted into BAM file format using SAMtools (v0.1.18). Initial mappings were postprocessed using Genome Analysis Toolkit (GATK) 1.3-21 following the “best practices V3.” In brief, reads were realigned around sites of known insertions and deletions (INDELs), and likely PCR duplicates were detected using Picard 1.48. Finally, raw base quality scores were empirically recalibrated. Single-nucleotide polymorphisms (SNPs) and short INDELs were identified using the UnifiedGenotyper from GATK and in parallel with the variant caller in SAMtools. Variants were classified as novel or known variants deposited in dbSNP 135. Functional consequences of each variant were annotated using snpEff 2.0.5d for UCSC hg19 RefSeq genes and ENSEMBL 65 human gene models. The potential deleterious effect was evaluated using PolyPhen 2, SIFT, PhyloP, MutationTaster, GERP++, LRT, and OMIM if available. Candidate variants from GATK and from SAMtools were compared to increase both sensitivity and specificity.

### Targeted next-generation sequencing panel.

A HaloPlex DNA targeted gene enrichment panel (Agilent Technologies) was designed using SureDesign software (www.agilent.com/genomics/suredesign) to capture all coding exons and 100 bp of intronic flanking sequence of genes of interest. The entire custom design covered 160 genes involved in adrenal development and function, for a total size of 497.956 kbp. The total number of amplicons was 21,612 with predicted target coverage of 98.7%. gDNA samples (225 ng each) were prepared for Illumina sequencing according to the HaloPlex Target Enrichment System protocol (version D.5, Agilent Technologies) (30). Libraries were sequenced on the Illumina MiSeq platform following the manufacturer’s instructions (Illumina). FAST-Q files were analyzed using SureCall (v3.0.1.4) software (Agilent Technologies).

### Variant analysis and validation.

To confirm the presence and identity of the variants, Sanger sequencing was performed on PCR amplicons from gDNA covering the variant position. Direct sequencing was carried out using Big Dye Terminator v1.1 Cycle Sequencing Kit (Applied Biosystems) on an ABI 3130 sequencer (Applied Biosystems) and visualized using Sequencher v5.2.4 (Gene Codes Corp.). Novel missense variants were absent in at least 100 control samples with adrenal insufficiency and normal birth weight and in population control databases such as the Exome Aggregation Consortium (ExAC) browser (Cambridge, Massachusetts, USA; http://exac.broadinstitute.org; accessed November 2015).

### Sequence alignment.

Multiple sequence alignments of SAMD9 were generated using Clustal Omega (www.ebi.ac.uk/Tools/msa/clustalo/).

### Quantitative reverse transcriptase PCR.

A human total RNA panel comprising several tissue sources (catalog 636643, Clontech Laboratories) was used to study the expression of *SAMD9*. The panel included brain, cerebellum, kidney, adrenal gland, lung, placenta, skeletal muscle, spleen, testis, thymus, uterus, fetal brain, and fetal liver. In addition, RNA from human fetal adrenal gland and testis (9 weeks postconception), provided with approval from the Human Developmental Biology Resource (HDBR, www.hdbr.org), was used. Purified RNA was quantified using a NanoDrop 1000 spectrophotometer (Thermo Fisher Scientific). RNA (2 μg) was reverse transcribed with the SuperScript III Reverse Transcription kit (Thermo Fisher Scientific) according to the manufacturer’s instructions. Quantitative reverse transcriptase PCR (qRT-PCR) was performed using the TaqMan Fast Advanced Master Mix (Applied Biosystems) and the SAMD9 TaqMan assay (Hs00539471_s1, Thermo Fisher Scientific), on the StepOnePlus System (Thermo Fisher Scientific). The relative expression of *SAMD9* was calculated as 2^–ΔΔCt^ using the comparative Ct (ΔΔCt) method and *GAPDH* (Hs02758991_g1, Thermo Fisher Scientific) or *ACTB* (Hs99999903_m1, Thermo Fisher Scientific) as internal housekeeping control. Experiments were repeated on 4 independent occasions, twice for each housekeeping gene. Representative data are shown for a single study performed with triplicate replicates.

For *SAMD9* mRNA expression in fibroblasts, samples were studied from 3 healthy control individuals and 3 patient samples (patients 4, 6, and 8). RNA was extracted from 4 × 10^6^ cells using the Trizol method. DNase digestion and cDNA synthesis were performed according to standard protocols (NEB, Promega). qRT-PCR reactions for *SAMD9* as well as the housekeeping genes *ACTB* and *GAPDH* were established according to standard protocols using an iCycler (Bio-Rad). Oligonucleotide sequences are shown in Supplemental Table 4. Results represent 4 independent experiments each performed in 2 replicates. The results were analyzed using the ΔΔCt method, and statistical analysis was performed using the Mann-Whitney test.

### Immunohistochemistry.

Human fetal adrenal gland and testis tissue (9 weeks postconception) was provided with approval from the HDBR. Following cryosection, tissue sections (12 μm) were fixed briefly in 4% paraformaldehyde in TBS, rinsed in TBS, and blocked in 1% BSA in TBS-Tween (0.5% Tween) for 1 hour before incubation overnight with mouse monoclonal anti–human SF-1 antibody (Invitrogen, N1665; 1:200 dilution) and rabbit polyclonal anti–human SAMD9 antibody (Sigma-Aldrich, HPA021319; 1:200 dilution). Sections were washed in TBS-Tween and incubated for 1 hour with the relevant secondary antibodies: Alexa Fluor 488–goat anti-mouse (Invitrogen, A11001; 1:400) and Alexa Fluor 555–goat anti-rabbit (Invitrogen, A21429; 1:400), respectively. Nuclei were counterstained with DAPI (Sigma-Aldrich). Slides were washed with TBS and mounted using ProLong Gold Antifade Mountant (Life Technologies). Images were collected on a Zeiss LSM 710 confocal microscope (Carl Zeiss) and analyzed using Zeiss Zen 2009 and ImageJ (NIH).

### Whole-genome array.

Chromosomal microarray copy number analysis was performed on gDNA and analyzed versus a reference (Affymetrix Reference Model file created from microarrays using 284 HapMap samples representing Yoruba, Asian, and Caucasian ethnic groups and 96 samples from phenotypically healthy males and females). The DNA was digested with Nsp1, amplified, fragmented to 25–125 bp, and biotin-labeled before hybridization to Affymetrix Cytochip 750K arrays in a GeneChip Hybridization Oven 645 in accordance with the manufacturer’s instructions (user’s guide; Cytoscan Assay Rev 4, Affymetrix). The microarrays were washed and stained in a GeneChip Fluidics Station 450 and scanned using a GeneChip Scanner 3000 (Affymetrix). Raw data were processed in Chromosome Analysis Suite v2 (Affymetrix) and examined using the high-resolution setting providing an estimated practical resolution of 200 kb.

### Single-nucleotide primer extension reactions.

Primers were designed to flank the specific patient mutations and sequence variants (Supplemental Table 5). After treatment with shrimp alkaline phosphatase and exonuclease I, PCR products were used in single-nucleotide primer extension reactions using the ABI Prism SNaPshot Multiplex kit (Applied Biosystems) and appropriate specific 35-mer forward primers to detect the nucleotide at the position of interest. After removal of unincorporated dideoxynucleotides (ddNTPs), the fluorescently labeled single-stranded DNAs were analyzed on the ABI 3730 sequencer (Applied Biosystems) together with the GeneScan 500 ROX dye size standard. Relative proportions of alleles were derived from the peak areas in the electropherograms. In pilot experiments, allele ratios were compared for PCR reactions conducted between 24 and 30 cycles to ensure that alleles were being independently amplified. For subsequent analyses, PCRs were run for 28 cycles.

### Subcloning studies.

Validation of low-copy-number variants and quantification of mutations were also performed by subcloning of DNA and screening of multiple independent colonies. In brief, gDNA from patients was PCR-amplified with a high-fidelity Taq polymerase, Elongase (Life Technologies). PCR products were ligated into the pCR-XL-TOPO vector and transformed into One Shot TOP10 chemically competent cells (Life Technologies). Plasmid DNA was extracted and the region of interest sequenced, as described above. Between 50 and 100 independent clones were studied in each experiment.

### Functional studies of SAMD9 WT and mutant activity.

A pCMV6-Entry vector containing human *SAMD9* cDNA was obtained from Origene (SC304503). All 7 single and 4 double SAMD9 mutations were introduced into the cDNA by site-directed mutagenesis using the QuikChange Multi Site-Directed Mutagenesis Kit (Agilent Technologies), and well as the 4 secondary changes alone (total *n* = 15). All vectors were sequenced to validate the mutations.

For functional studies of cell proliferation, 2 × 10^5^ HEK293 cells (ATCC CRL-1573) were transfected with the pCMV6-Entry vector with the 17 different SAMD9 variants (15 mutants, WT, or empty vector) using Lipofectamine 3000 (Thermo Fisher Scientific) following the manufacturer’s protocol. All cells were negative for mycoplasma. Stably transfected cells were selected with G418 (500 μg/ml) (Gibco) for 14 days and collected once the controls reached confluence. Representative images of cell density were taken by bright-field microscopy using an Olympus 1X71 inverted microscope. Cells were trypsinized, pelleted, and resuspended before being counted on a FACSCalibur sorter (BD Biosciences) using propidium iodide to exclude dead cells. Experiments were performed in triplicate on 3 independent occasions. Results are presented as mean (SD) for all 3 studies. These studies were all performed in a blind fashion by the investigator.

### Fibroblasts.

Skin fibroblasts from patients 4, 6, and 8 and from 2 controls were grown in DMEM supplemented with 10% FBS and 1% penicillin/streptomycin at 37°C in a humidified atmosphere (5% CO_2_). All fibroblast cultures used were negative for mycoplasma contamination.

### Western blot analysis.

Total protein lysates from fibroblast cultures (passage 2–5; 4 μg) were transferred with a semidry electrophoretic method according to standard protocols on a PVDF membrane (PerkinElmer). The membrane was blocked with 5% nonfat dry milk in 0.03% PBS-Tween and sequentially incubated with anti-SAMD9 (Sigma-Aldrich, HPA021319) and anti–α-tubulin Clone B-5-1-2 (Sigma-Aldrich, T5168) antibodies. As a second antibody, polyclonal goat/anti-rabbit IgG–HRP (DAKO, P0448) and anti-mouse IgG–peroxidase (Roche, 12015218001) were used. Bands were visualized with lumi-light plus Western blotting substrates (Roche). Four independent Western blotting experiments were performed.

### Assay for cell proliferation.

Fibroblast proliferation was evaluated using a Cell Proliferation ELISA BrdU colorimetric kit (Roche Diagnostics). Cells were cultured in DMEM supplemented with 0.2% FBS and 1% penicillin/streptomycin at 37°C in a humidified atmosphere (5% CO_2_) for 6 days. Thereafter, 1.5 × 10^4^ cells were seeded in 96-well plates, and after 24 hours BrdU solution (10 μM) was added and cells were incubated for an additional 24 hours. The incorporation of BrdU into the DNA was quantified according to the manufacturer’s protocol. Three independent experiments were performed, each with 6 technical replicates, and 1-way ANOVA with Tukey’s multiple comparison test was used to compare proliferation of patient cells against controls. For 1 experiment, we failed to obtain reads for control 1 and 2 technical replicates for patients 4 (sample 2) and 6; therefore the number of replicates for those samples is different (control 1, *n* = 12; patients 4 and 6, *n* = 16).

### Electron microscopy.

The cellular ultrastructural morphology of cultured skin fibroblasts was examined from 3 affected individuals and 2 controls. Cells were pelleted, fixed in 2.5% glutaraldehyde followed by 1% osmium tetroxide, and then processed into agar 100 resin. Sections (90 nm) were cut and stained with uranyl acetate and lead citrate. Ultrathin sections were examined using a JEOL 1400 transmission electron microscope (JEOL). Independent images from 20 randomly selected control or patient fibroblasts were studied.

### Endosome studies.

Fibroblasts were grown on a sterile tissue culture dish with a cover glass bottom (FD35-100, FluoroDish, World Precision Instruments) and were cotransfected with the RFP-Rab5a–expressing vector and the GFP-Rab7a–expressing vector (CellLight Late Endosomes-GFP, Life Technologies). Live cells were imaged 48 hours after transfection using a Zeiss LSM 710 confocal microscope (Carl Zeiss) and analyzed using Zeiss Zen 2009 and ImageJ (NIH). For quantitative studies of early endosome volume, fibroblasts were cotransfected with the RFP-Rab5a vector as above, and after 48 hours culture medium was removed and cells were washed with PBS. Cells were fixed for 20 minutes with 4% paraformaldehyde in PBS at room temperature, and then washed 3 times with PBS. Ten images/cells per patient and control were collected using a Zeiss LSM 710 confocal microscope (Carl Zeiss). Deconvolution of raw images was carried out using Huygens Software (Scientific Volume Imaging); endosome volume was measured using Imaris Software (Bitplane). Statistical analysis was performed in Prism (GraphPad), using 1-way ANOVA and Tukey’s post hoc test (*P* < 0.05). These studies were done in a blind fashion by the investigator.

### Reprogramming.

Reprogramming of patient and control fibroblasts was performed using Sendai virus vectors (CytoTune-iPS 2.0 Reprogramming Kit, Thermo Fisher Scientific). In brief, 5 × 10^4^ cells were seeded in 1 well of a 24-well plate and cultured in proliferation medium that consisted of DMEM supplemented with 10% FCS, 1× GlutaMAX with 100 U/ml penicillin, and 100 μg/ml streptomycin (all from Thermo Fisher Scientific). Cells were transduced using the 3 Sendai polycistronic vectors KLF4–OCT3/4–SOX2, c-Myc, and KLF4 (CytoTune-iPS 2.0 Sendai Reprogramming Kit, Thermo Fisher Scientific). After addition of the virus, the plate was centrifuged at 800 *g* for 20 minutes at room temperature. On the next day, medium was exchanged with fresh proliferation medium. Cells were cultured for 7 days with medium changes every other day. On day 8 after transduction, cells were passaged using TrypLE Select (Thermo Fisher Scientific) and seeded with a density of 3 × 10^4^ cells per well in proliferation medium onto a 6-well plate coated with Geltrex (Thermo Fisher Scientific). On the next day, medium was changed to Essential 8 (E8) medium (Thermo Fisher Scientific) supplemented with 100 U/ml penicillin and 100 μg/ml streptomycin. The E8 medium was replaced every day until human iPSC colonies appeared. Individual colonies were picked using a pipette tip and expanded in E8 medium on plates coated with Geltrex. Cell lines generated from the 2 patients are named CUBi001-A (patient 6) and CUBi002-B (patient 8). Detailed information on the generated cell lines is available in the Human Pluripotent Stem Cell Registry (https://hpscreg.eu/).

### Culture of iPSCs.

The iPSCs were cultured in E8 medium supplemented with 100 U/ml penicillin and 100 μg/ml streptomycin in 6-well dishes coated with Geltrex and routinely passaged using 0.5 mM EDTA (Thermo Fisher Scientific) in PBS (without calcium and magnesium) every 5–7 days at a splitting ratio of about 1:20. The iPSC lines BIHi001-A (control 1) and BIHi004-A (control 2) (both lines derived from fibroblasts of healthy donors) were used as controls in the differentiation and gene expression analysis experiments. Details of these lines are available at https://hpscreg.eu/.

### Immunofluorescence staining for pluripotency markers.

For staining, iPSCs were grown in 96-well imaging plates (CellCarrier, PerkinElmer) in E8 medium. After 3 days, cells were fixed with Cytofix reagent, followed by blocking and permeabilization with PermWash reagent (both from BD Biosciences). Then, cells were incubated with the dye-conjugated antibodies anti–TRA-1-60–Vio488 (Miltenyi Biotec, REA157, 130-106-872), anti-SSEA4–PerCP-Vio700 (Miltenyi Biotec, REA101, 130-105-053), anti-OCT3/4 (Isof. A)–APC (Miltenyi Biotec, REA338, 130-105-555), and anti-NANOG (D73G4)–PE (Cell Signaling Technology, 14955). Nuclei were stained with Hoechst 33342 (2.5 μg/ml in PBS; Invitrogen). Images were captured using an Operetta high-content imaging system (PerkinElmer).

### Karyotyping of iPSCs.

Karyotyping was carried out using KaryoLite BoBs (PerkinElmer). From each sample at least 240 ng of gDNA was used as a starting material. The samples were processed according to the manufacturer’s instructions. Briefly, gDNA was labeled with biotin, purified, and hybridized to beads with complementary BAC probes. After washing and reporter (streptavidin-PE) binding, the fluorescent signals were measured with a Bio-Plex 200 system (Bio-Rad). Results were analyzed using BoBsoft analysis software (PerkinElmer). As references, female and male gDNA (Promega) was used. A sample was defined as “normal disomic” when the fluorescent ratio was approximately 1.0 for all loci analyzed.

### Differentiation of iPSCs into intermediate mesoderm cells.

iPSCs were differentiated into intermediate mesodermal cells using a protocol described by Lam et al. (31). iPSCs were harvested as single cells using Accutase (Thermo Fisher Scientific) and 4 × 10^5^ cells seeded in each well of a Geltrex-coated 6-well plate in E8 medium. Medium was changed after 3 days to advanced RPMI, 1× GlutaMAX (both from Thermo Fisher Scientific), 5 μM CHIR99021 (Reagents Direct), 100 U/ml penicillin, and 100 μg/ml streptomycin for 36 hours. For the next 72 hours, the differentiation medium was changed to advanced RPMI, 1′ GlutaMAX, 100 ng/ml bFGF (PeproTech), 2 μM retinoic acid (Miltenyi Biotec), 100 U/ml penicillin, and 100 μg/ml streptomycin. The cells were harvested using Accutase, and total RNA for gene expression analysis was extracted as described above.

### Expression profiling.

Total RNA was isolated using the RNeasy Kit (Qiagen) from the 2 patient iPSC lines (patients 6 and 8) and the control iPSC line (control 1), as well as from the primary fibroblasts these lines were derived from. In addition, RNA was isolated from the 2 patient intermediate mesoderm cell lines (patients 6 and 8) and 2 control intermediate mesoderm cell lines (controls 1 and 2). Biotin-labeled cRNA was generated using the Illumina TotalPrep RNA Amplification Kit (Ambion, USA) with 300 ng of quality-checked total RNA as input. cRNA samples were hybridized on HumanHT-12 v4 Expression BeadChips (Illumina). Data analysis was performed using Genome Studio Software v2011.1 (Illumina) and Bioconductor (32). Functional enrichment analysis was performed using DAVID (http://david.abcc.ncifcrf.gov). Validation of pluripotency was carried out via the Web-based PluriTest open access software, a bioinformatics assay of pluripotency in human cells based on gene expression profiles (33).

### Statistics.

Statistical analysis was performed using GraphPad Prism and is described in the relevant sections. Student’s *t* tests were 2-tailed. A *P* value less than 0.05 was considered significant. Microarray data were analyzed using Bioconductor.

### Data availability.

All relevant data are available from the authors upon request. Microarray data are available through ArrayExpress (https://www.ebi.ac.uk/arrayexpress/) (accession code E-MTAB-5145).

### Study approval.

Written informed consent of the parents was obtained for research and diagnosis prior to inclusion in the study (UK IRB 07/Q0508/24; HDBR 08/H0712/34+35).

## Author contributions

FB and PK designed studies, conducted and supervised data generation and analysis, and provided input in the manuscript preparation. JPS, IDV, M Digweed, HS, NK, WC, GKK, GA, DM, and DAM conducted data generation and analysis and were involved in study design. M Didi, AFB, OB, AMP, PD, JKHW, PG, DK, and BS all provided clinical input, samples, and discussions about the phenotypes. SAM, ME, MWW, and CMN contributed to the interpretation of the mechanisms leading to the emerging hematological phenotype or disease mechanisms and provided clinical data and samples. AG and JCA designed the studies, supervised data generation, interpreted the results, and wrote the manuscript.

## Supplementary Material

Supplemental data

## Figures and Tables

**Table 1:**
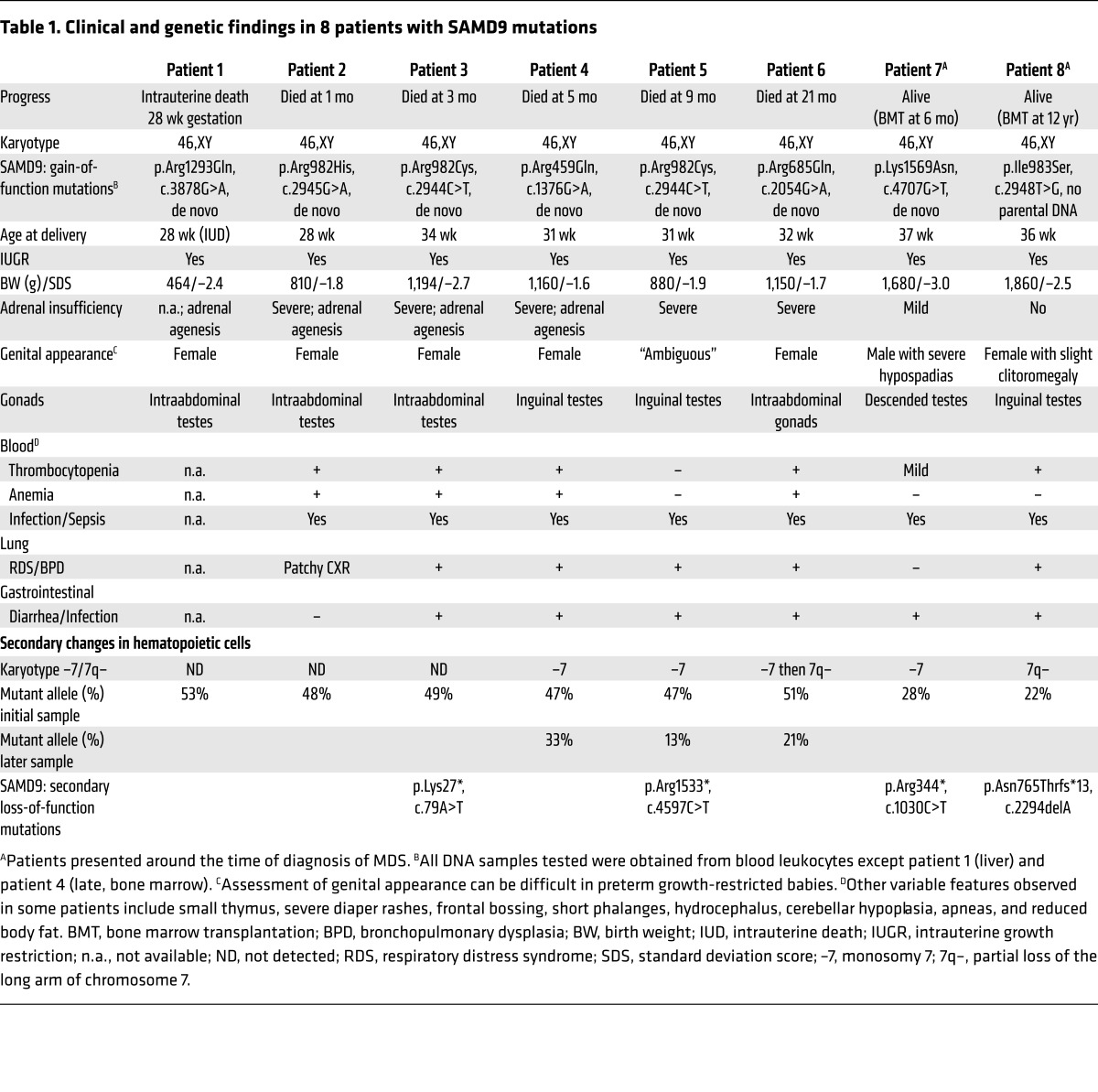
Clinical and genetic findings in 8 patients with SAMD9 mutations
