# Supplementary material for: Somatic mutations and progressive monosomy modify SAMD9-related phenotypes in humans
Source: J Clin Invest. 2017 Mar 27;127(5):1700–13. doi: 10.1172/JCI91913 (PMC5409795; doi:10.1172/JCI91913)
Supplement: Supplemental data [file jci-127-91913-s001.pdf]

Supplemental Data

Dynamic genomic changes rescue severe SAMD9-related growth restriction in humans

Supplemental Table 1. Endocrine data (testis, adrenal)

Patient	Age at delivery	Age at sample (chronological)	Testosterone (ng/dl)	AMH (ng/ml)	Cortisol (µg/dl)	ACTH (pg/ml)	Interpretation
3	34 weeks	2 weeks	<10	-	<0.4	-	Adrenal and Leydig cell insufficiency
4	31 weeks	19 days	106	-	<1.8	>1250	Adrenal and Leydig cell insufficiency
		34 days	106 [A]	-	-	-	
5	31 weeks	8 days	<20	-	5.0 (2.7 to 2.9) [C]	>5000	Adrenal and Leydig cell insufficiency
		5 weeks	250 (post hCG) [B]	-	-	-	
		2 months	92	-	-	-	
6	32 weeks	3 days	2.9 [A]	-	0.2	-	Adrenal and Leydig cell insufficiency, normal Sertoli cell function
		10 days	5.8 [A]	23	0.2	>1000	
		3 weeks	14 [A]	-	-	-	
		2 months	40 [A]	-	-	-	
		3 months	35 [A]	-	-	-	
7	37 weeks	6 days	115	-	9.1	82	Partial adrenal and Leydig cell insufficiency
		18 days	-	-	5.1	114	
		7 weeks	389	-	-	-	

[A] Tandem Mass Spectrometry assay instead of immunoassay; [B] post twice weekly stimulation with human chorionic gonadotropin (hCG) for 3 weeks; [C] basal and peak cortisol in cosyntropin stimulation test.

Conversions: testosterone ng/dl to nmol/l, multiply by 0.0347; AMH ng/ml to pmol/l, multiply by 7.14; cortisol µg/dl to nmol/l, multiply by 27.6; ACTH pg/ml to pmol/l, multiply by 0.22.

Normal ranges can be dynamic and influenced by preterm delivery. Basal testosterone typically falls in the first 5 days after delivery and then rises between 50-90 days in the “minipuberty of infancy” to peak around 300-450 ng/l. The timing of the testosterone rise in preterm infants may be delayed compared to term babies. AMH concentration in first 2 weeks of life is typically 30 ng/ml. Cortisol basal values are typically 5-14 µg/dl and peak response to cosyntropin >20 µg/dl. ACTH normal range 10-60 pg/ml.

Note: immunoassays can be unreliable in preterm infants because of cross reactivity with fetal adrenal steroids, although in these children the fetal adrenal output was generally low.

Supplemental Table 2. Autopsy findings

Patient	Age at delivery	Age at death (chronological)	Adrenal findings at autopsy	Testis findings at autopsy
1	28 weeks	Intrauterine	No adrenals found	Testes with tubules
2	28 weeks	1 month	No adrenals found	Tubular structures consistent with testes, few Leydig cells
3	34 weeks	3 months	No adrenals found	Hypoplastic testes with fibrosis
4	31 weeks	5 months	Occasional islands of adrenal tissue	Normal testicular tissue with fewer Leydig cells

Supplemental Table 3. Functional enrichment analysis using DAVID

	Fibroblasts	ES	Patient derived iPSC	ES	iMesoderm	ES
downregulated	Urogenital development	2.58	Urogenital development	6.08	Steroid biosynthesis	3.7
	EGF pathway	2.41	Transcription	3.88	Transcription	3.38
	Sequence specific binding	2.14	Sequence specific binding	3.68	Lung development	3.32
upregulated	Cell cycle	15.21	EGF/IGF	7.94	Inflammation	7.41
	Embryonic development	3.92	Embryonic development	5.77	EGF/IGF pathway	6.3
	IGF pathway	1.9	Sequence specific binding	4.62	Apoptosis	3.69

ES, enrichment score; iPSC, induced pluripotent stem cell; EGF, epidermal growth factor; IGF, insulin-like growth factor.

Supplemental Table 4. qRT-PCR primer sequences

Gene	Primer sequence
SAMD9	F CAGGACCAGGCAATCTCATT<br>R ATCTCCACTCTGCCACAAGG
GAPDH	F AGAAGGCTGGGCTCATTTG<br>R AGGGCCATCCACAGTCTTC
ACTB	F CTGGGACGACATGGAGAAAA<br>R AAGGAAGGCTGGAAGAGTGC

Supplemental Table 5. Primer sequences for single nucleotide primer extension assays

Patient	Mutation/variant	Flanking primers	Detection primer
1	c.1376G>A	F AAATAAATGCCACCCAGATC<br>R TCCACAGAGGACAGTAATAG	CATCAATGGAGTGGTCAAAGCTTACAAAGAAAGCC
2	c.2054G>A	F AGCCTTGAAGAGATCAATGG<br>R AAACTGGTTACCTGTTCTCC	AAAAATCAAGGCATCAAAAGAGGAAGACTTCTATC
3 and 4	c.2944C>T	F ATGTGGTCCGGAATATCCTG<br>R ATGTTCATCGCGGTGTCTTG	CAGAGGTCATCGAATGTGGGAACTACTGTGGAGTA
3	c.4597C>T	F AAGGAAAAAATTGACCAGTGC<br>R TGGGTACTGAGATCAAATTC	AAAAAGTCCAAGAACTTTTGCTTCGTTTACAAGGT
5	c.2945G>A	F ATGTGGTCCGGAATATCCTG<br>R ATGTTCATCGCGGTGTCTTG	AGAGGTCATCGAATGTGGGAACTACTGTGGAGTAC
6	c.2948 T>C	F ATGTGGTCCGGAATATCCTG<br>R ATGTTCATCGCGGTGTCTTG	GGTCATCGAATGTGGGAACTACTGTGGAGTACGCA
6	c.2294_2294delA	F AGCCTTGAAGAGATCAATGG<br>R AAACTGGTTACCTGTTCTCC	ACTAAGGAAGAAATTCAGATGTGCTGTGCTGAAAA
7	c.3878G>A	F AAGTAGTGATATTCCAGGGG<br>R TGACTGGATTTTGACAGTGC	GAACAATATTAAGCAAAATGAAGAGGCCAAAACTC
8	c.4707G>T	F AAGGAAAAAATTGACCAGTGC<br>R TGGGTACTGAGATCAAATTC	TTAGGTCAACTTAGAAGTGGCAGAAGCATAGAGAA
8	c.1030C>T	F ATCAAGTCCAACAAGCAAAG<br>R GTTACAAGAATGTACTGTTC	TATGGGAACAAAGTAAAAAATTCTCACTATTTGTG

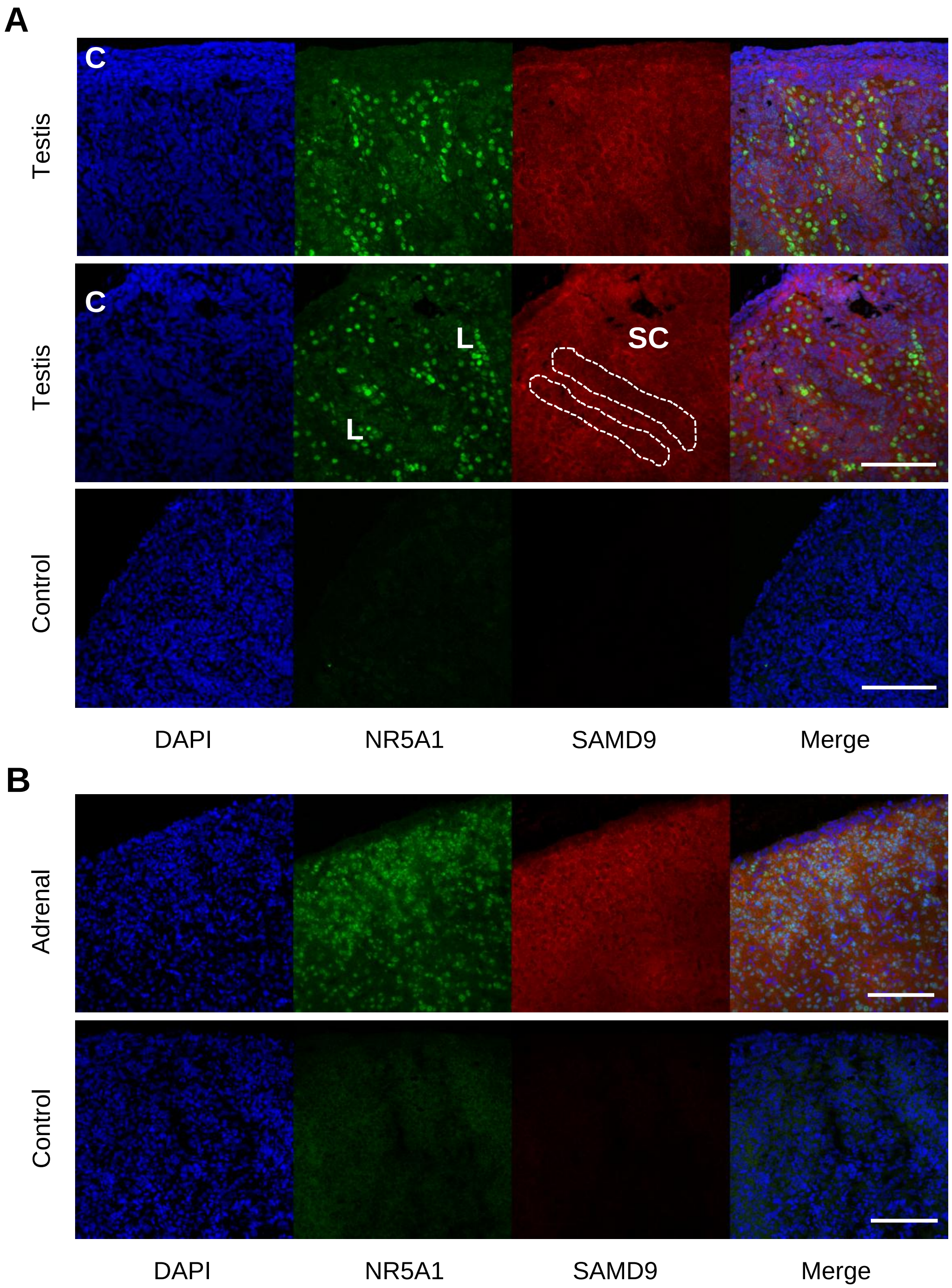

Supplemental Figure 1. SAMD9 is expressed in human fetal testis and adrenal gland. (**A**) Immunohistochemistry showing SAMD9 expression in human fetal testis at 9 weeks post conception (red) with NR5A1 (also known as steroidogenic factor-1) shown in green and DAPI-stained nuclei in blue. Control data without the primary antibody are shown below. The thin layer of DAPI-stained cells is the capsule (C). NR5A1 highlights the nuclei of interstitial Leydig cells (L). SAMD9 appears in the cytoplasm of both interstitial cells and cells in the seminiferous cords (SC). Scale bar, 100μm. (**B**) Control data for the fetal adrenal immunohistochemistry (Figure 2A). There is some autofluorescence of the cytoplasm of adrenal cells in the green channel, but the pattern is distinct from the nuclear staining of NR5A1. Scale bar, 100μm.

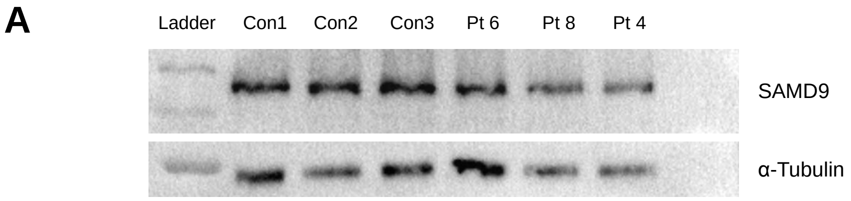

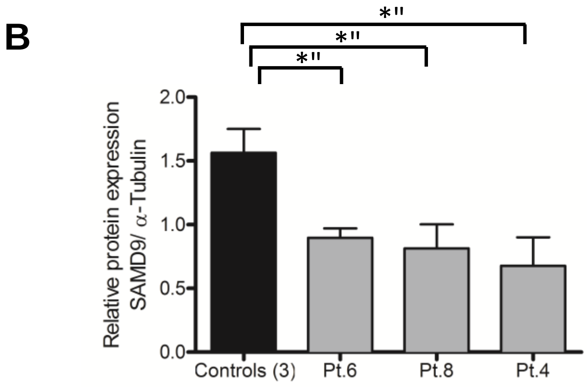

Supplemental Figure 2. SAMD9 protein expression was analyzed by Western blot analysis in fibroblast samples from Patients 4, 6 & 8 and compared to the expression of α-tubulin as a control. The results were compared with three control fibroblast samples from healthy individuals. (**A**) Example of a representative Western blot. (**B**) Protein expression analysis was performed based on the data of four independent experiments. Data are shown as mean ± SEM (* *P*<0.05, unpaired t-test).

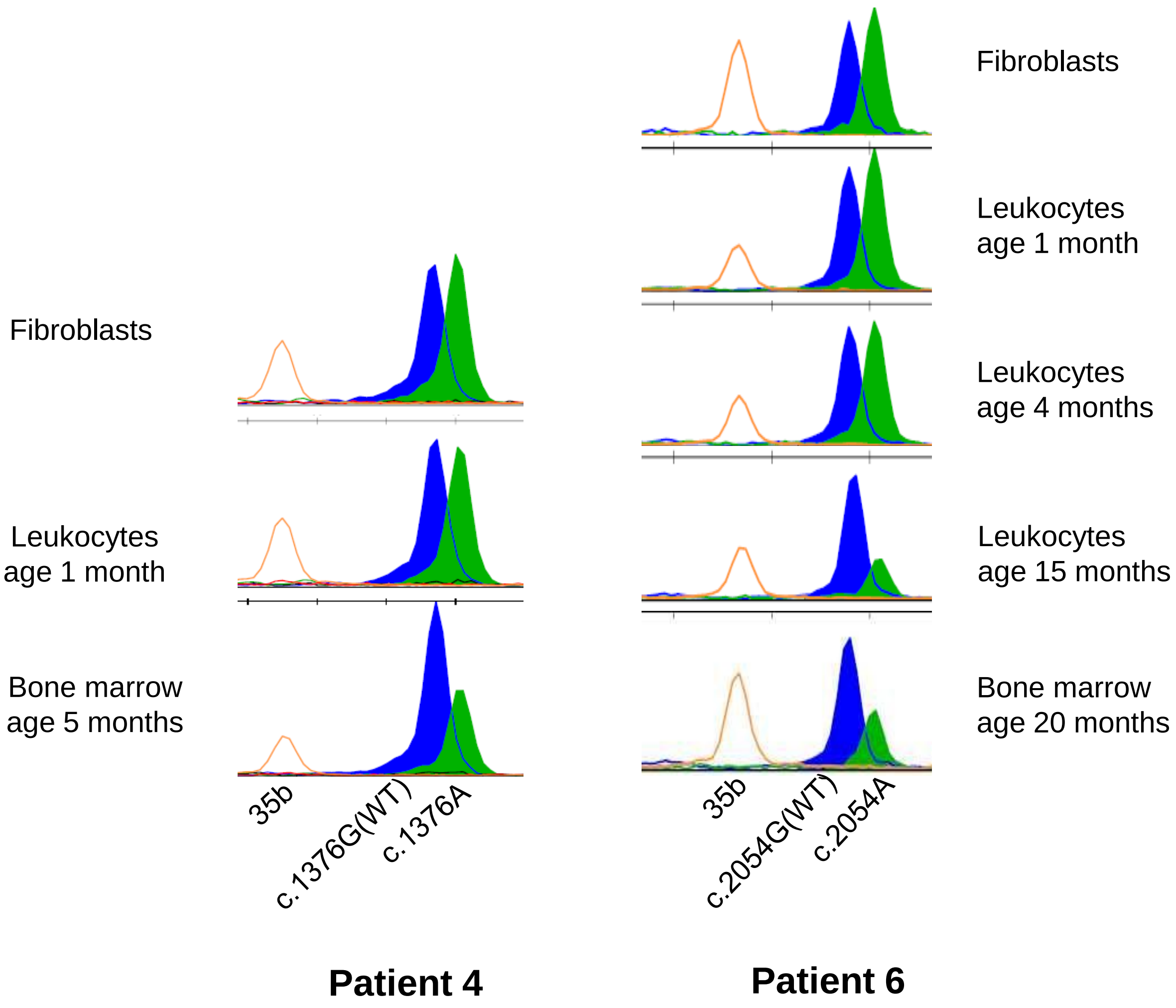

Supplemental Figure 3. SAMD9 allele distribution in serial samples from Patients 4 & 6. Electropherograms of single nucleotide primer extension reactions in genomic DNA are shown from the samples described. PCR products were generated to flank the c.1376G>A or c.2054G>A mutation and single nucleotide extension from a specific 35mer primer was used to probe the nucleotides at these positions. Fragments were separated on the ABI-3730 sequencer. The position of a 35b marker is indicated.

A

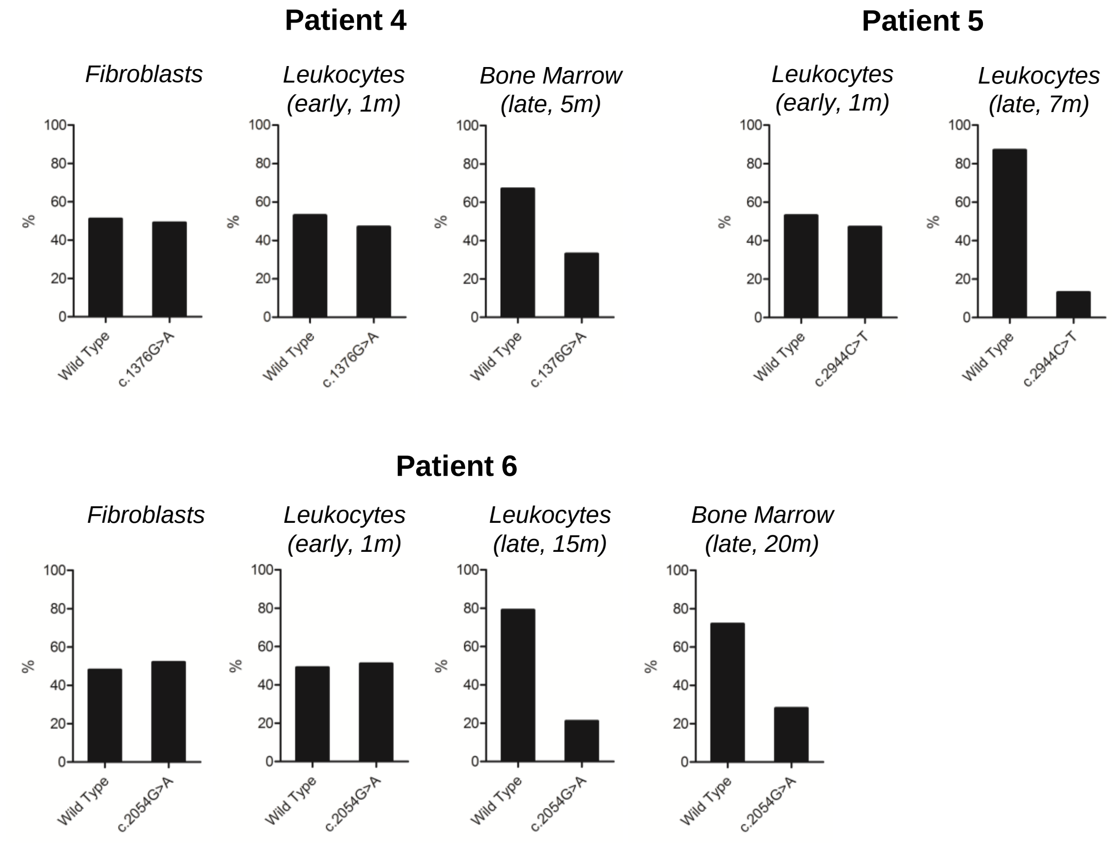

B

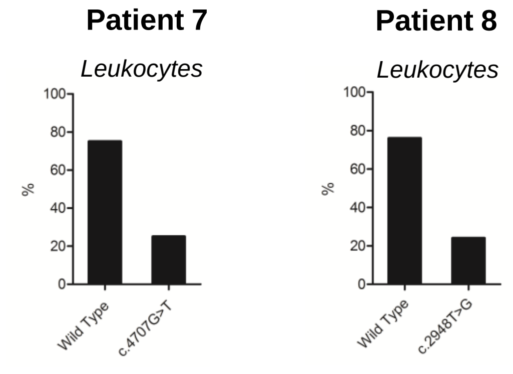

Supplemental Figure 4. Changes in the wild type and mutant allele percentage in different tissues and over time to show a selective reduction of the mutant *SAMD9* allele with the development of monosomy. (**A**) Serial data from the three children with acquired monosomy. The allele percentages were determined using single-nucleotide primer extension assays. (**B**) Blood leukocytes from the two patients who had early monosomy 7 and myelodysplasia. Allele percentages were determined by PCR amplification and subcloning of DNA followed by sequencing of more than 50 independent clones.

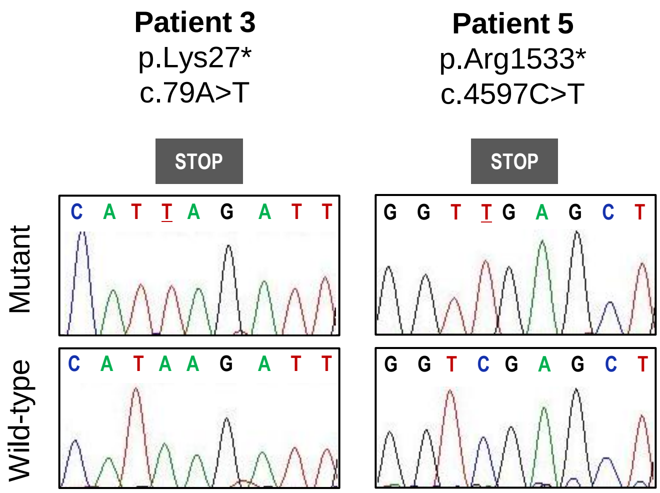

Supplemental Figure 5. Low-level nonsense mutations detected in Patients 3 and 5 on deep sequencing were confirmed by subcloning. Mutant clones are shown above (mutant nucleotide underlined) and wild-type clones are shown below.

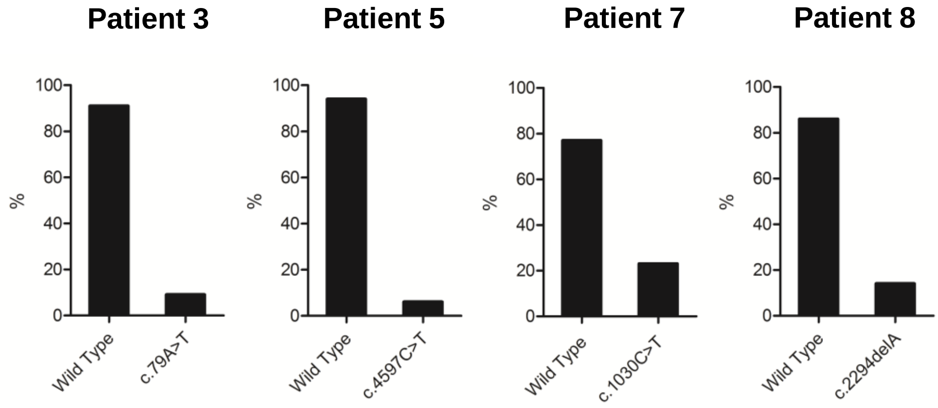

Supplemental Figure 6. Confirmation of additional loss-of-function nonsense and frameshift changes following subcloning of PCR-amplified DNA from Patients 3, 5, 7 and 8. Parental DNA was also subcloned for these regions (Patients 3, 5 and 7) and did not show any mutations.

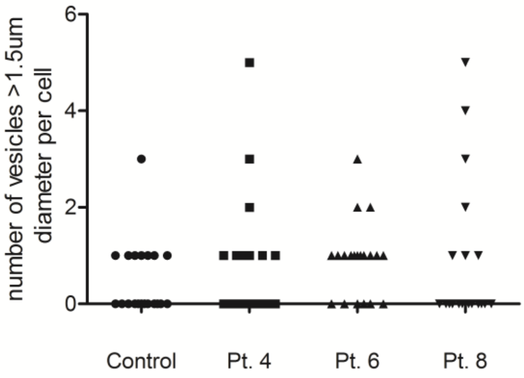

Supplemental Figure 7. Number of vesicles greater than 1.5 μm diameter per cell. Data are presented for 20 individual fibroblasts from each patient or control studied by electron microscopy. No statistically significant differences were seen between groups.

Patient 6
p.Arg685Gln
c.2054G>A

Probe	/F	/M
01C	1,00	0,95
02C	1,03	1,00
03C	1,01	1,00
04C	0,97	0,92
05C	0,97	0,94
06C	0,88	0,92
07C	0,97	1,01
08C	1,03	1,02
09C	1,04	1,04
10C	0,97	1,01
11C	0,99	0,95
12C	1,04	1,03
13C	0,93	0,95
14C	1,00	0,99
15C	0,93	0,99
16C	1,06	1,03
17C	1,00	0,95
18C	1,00	0,96
19C	1,09	0,99
20C	1,02	0,97
21C	1,01	1,01
22C	0,99	0,95
Auto	1,05	1,04
X1;ptel	0,63	0,89
X2;pcen	0,55	0,77
X3;qcen	0,67	0,95
X4;qtel	0,64	0,90
Y1;ptel	1,88	0,99
Y2;pcen	3,29	0,89
Y3;qcen	1,50	0,98
Y4;qtel	2,50	1,10

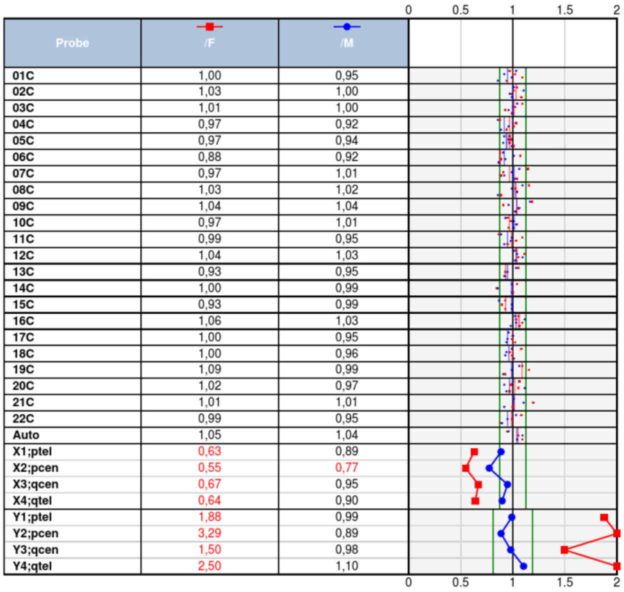

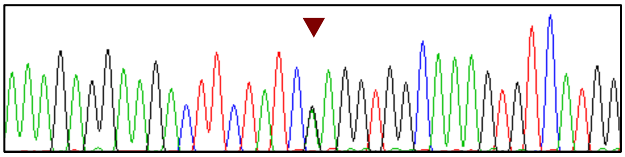

Patient 8
p.Ile983Ser
c.2948T>G

Probe	/F	/M
01C	1,06	1,00
02C	1,00	1,04
03C	0,95	1,00
04C	1,05	1,07
05C	0,96	1,04
06C	1,04	1,00
07C	1,01	1,02
08C	0,91	0,97
09C	0,90	1,00
10C	0,90	0,87
11C	1,16	1,11
12C	0,99	0,97
13C	0,92	1,02
14C	0,91	0,94
15C	1,01	1,10
16C	0,94	0,99
17C	1,02	0,99
18C	0,92	0,91
19C	1,17	1,01
20C	0,99	1,01
21C	1,09	1,07
22C	0,96	0,88
Auto	1,05	1,07
X1;ptel	0,74	1,04
X2;pcen	0,66	1,04
X3;qcen	0,65	0,87
X4;qtel	0,76	1,00
Y1;ptel	1,60	1,13
Y2;pcen	3,31	1,01
Y3;qcen	1,73	1,14
Y4;qtel	2,50	0,88

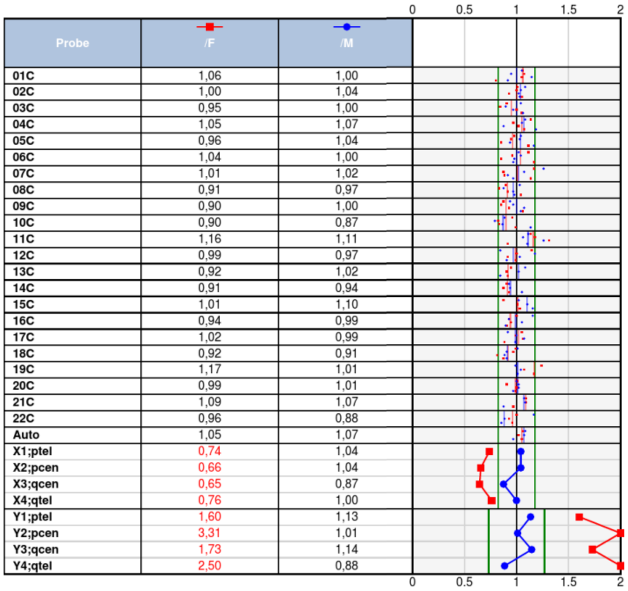

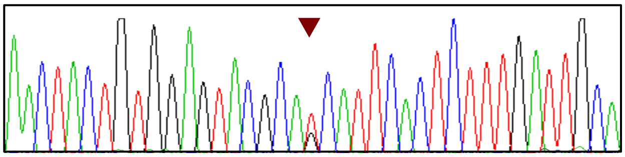

Supplemental Figure 8. iPS cells generated from Patients 6 & 8 were karyotyped and sequenced in order to confirm no large chromosomal changes had occurred and that the primary SAMD9 mutation was present.
